# Metaphysical indeterminacy in Everettian quantum mechanics

**DOI:** 10.1007/s13194-023-00562-5

**Published:** 2024-01-04

**Authors:** David Glick, Baptiste Le Bihan

**Affiliations:** 1 Department of Philosophy, University of California, Davis, 1 Shields Avenue, Davis, CA 95616 USA; 2 Department of Philosophy, University of Geneva, 2 Rue de Candolle, 1205 Geneva, Switzerland

**Keywords:** Many, Worlds, Everett, Quantum, Mechanics, Metaphysical, Indeterminacy

## Abstract

The question of whether Everettian quantum mechanics (EQM) justifies the existence of metaphysical indeterminacy has recently come to the fore. Metaphysical indeterminacy has been argued to emerge from three sources: coherent superpositions, the indefinite number of branches in the quantum multiverse and the nature of these branches. This paper reviews the evidence and concludes that those arguments don’t rely on EQM alone and rest on metaphysical auxiliary assumptions that transcend the physics of EQM. We show how EQM can be ontologically interpreted without positing metaphysical indeterminacy by adopting a deflationary attitude towards branches. Two ways of developing the deflationary view are then proposed: one where branches are eliminated, and another where they are reduced to the universal quantum state.

## Introduction

The contemporary version of *Everettian quantum mechanics* (EQM) holds that the fundamental description of reality is given by the universal quantum state, which evolves unitarily according to Schrödinger’s equation. When there is sufficient decoherence, this fundamental quantum state description gives rise to a plurality of quasi-classical branches (Saunders, 2010; Wallace, 2012). The universal quantum state generically takes the form of a massive superposition of states corresponding to the values of physical quantities, seemingly describing a reality of widespread indeterminacy. The key move for the Everettian is to substitute this indeterminacy in the values of physical quantities with a multiplicity of branches or worlds, each with determinate values of the quantities in question.^1^ Hence, a single indeterminate reality is replaced with a multiplicity of determinate branches, each of which can be thought of as a well-defined region of reality. As A. Wilson sums up the situation, “the central idea of [Everettian quantum mechanics] is to replace indeterminacy with multiplicity” (2020, p. 77).^2^

Recently, this narrative has been challenged by those seeking evidence of metaphysical indeterminacy in quantum theory. There are two broad areas where indeterminacy may slip back into the Everettian picture. First, only certain superpositions are amenable to the multiplicity solution, namely those which have undergone environmental decoherence, which appears to be a highly localised process. Indeterminacy thus seems to remain fundamentally present in states of affairs described by *coherent* superpositions (i.e., superpositions which have yet to undergo decoherence). Second, even if indeterminacy is not found in the fundamental description provided by the universal quantum state, it could still emerge at a less fundamental level of description, which corresponds to the multiplicity of branches. The number and nature of Everettian branches would thus appear to be instances of metaphysical indeterminacy not found at the fundamental level. Thus, these provide putative examples of derivative metaphysical indeterminacy.

Our primary aim in this paper is to respond to such arguments by offering an understanding of EQM free from metaphysical indeterminacy. We maintain that one can regard reality as perfectly determinate, and relegate any residual indeterminacy in our understanding of reality to the representational realm. Ultimately, the question of derivative indeterminacy in EQM turns on how we understand the inter-level links connecting the fundamental and emergent levels. As a secondary objective, we will urge that a deflationary understanding, which eschews metaphysical indeterminacy, is the most plausible one. Note that this second objective, which consists in motivating the deflationary understanding as the most plausible, is secondary to our primary objective, which is to show that EQM alone does not motivate the endorsement of metaphysical indeterminacy. The very existence of a plurality of possible interpretations of EQM is sufficient to assert our primary objective, namely to establish that EQM alone does not imply the existence of metaphysical indeterminacy—and, this, independently of whether we also succeed in motivating the deflationary approach as being best. In the end, it should be clear that the existence of metaphysical indeterminacy in the setting of EQM results from metaphysical commitments (e.g., the interpretation one adopts toward the inter-level links involved between the two levels) rather than from EQM alone.

Before we proceed, it is worth placing our discussion in a broader context. Much of the discussion of quantum indeterminacy assumes that quantum mechanics is in fact theoretically fundamental, at least regarding the aspects that are relevant to the debate, and thus that it bears on fundamental metaphysics. Naturally, this is an important simplification, as non-relativistic quantum mechanics, quantum field theories and semi-classical gravity are generally considered to be provisional theories, which are—at best—partially correct. Only with a final, absolutely fundamental theory could one be justified in moving from a fundamental description given by such a theory to a fundamental description of reality itself (McKenzie, 2021). The question of whether it is possible to formulate an absolutely fundamental theory—a *theory of quantum gravity* that is final—and whether its ontology will preserve knowledge about determinacy and indeterminacy acquired through the study of non-relativistic quantum mechanics is of course a delicate matter.^3^

Our less ambitious task here is to assess whether there is metaphysical indeterminacy according to EQM. We take no position on whether the status of ‘fundamental’ indeterminacy according to EQM would carry over to any relatively more fundamental physical theories and, a fortiori, to any final theory.

Our discussion proceeds as follows. In the next section, we consider the case for fundamental indeterminacy in EQM. Our claim there is that EQM fails to provide any novel motivation for fundamental metaphysical indeterminacy. In Section 3, we turn to derivative forms of indeterminacy in EQM. In particular, we outline the apparent indeterminacy in the number and nature of Everrettian branches discussed by A. Wilson (2020) and Calosi and J. Wilson (2022). Section 4 argues that the existence of derivative metaphysical indeterminacy in EQM turns on the nature of the *inter-level links* deployed in EQM. We argue that a deflationary link better aligns with standard physical reasoning and the aims of EQM. We go on to distinguish between two deflationary approaches—eliminativism and reductionism—in Section 4.3. Our conclusion is that, on either deflationary approach, there is no clear basis for derivative metaphysical indeterminacy in EQM. Combined with the conclusion of Section 2, this means that EQM does not provide any new motivation for metaphysical indeterminacy.

## Fundamental indeterminacy

The fundamental description of reality provided by EQM is just the *bare theory* (Albert, 1994). At each moment, there is a perfectly well-defined universal quantum state (which may be represented by a wavefunction) which evolves unitarily according to Schrödinger’s equation (or its relativistic analogue). There is considerable debate over the metaphysics of fundamental reality on EQM; the correct formal representation of the quantum state, the status of spacetime, and other issues remain controversial. But it seems clear that, however it’s understood formally, the universal quantum state is fully determinate and the law that governs its evolution is fully deterministic. It thus seems that there is no place for indeterminacy at the fundamental level in EQM.

However, Peter Lewis recognizes a kind of fundamental indeterminacy that may be present in EQM:

Fundamental particles like electrons typically lack determinate values for their physical properties. This indeterminacy...is quite different from that typically countenanced in the philosophical literature on the subject, because it has nothing to do with composition or with the familiar kind of vagueness. (Lewis , 2016, p. 88)

So, it would seem that even if the quantum state is perfectly determinate, the values of physical quantities for individual objects may engender indeterminacy. And, given that some of the objects are elementary, such indeterminacy may be viewed as *fundamental*. The first thing to note is that there are different notions of fundamentality, with different extensions. Thus, the universal quantum state may be fundamental in the sense of offering a description that grounds all other descriptions, while elementary particles may be fundamental in the sense of lacking proper physical parts. That said, perhaps leaving a description of fundamental reality at the level of the universal quantum state isn’t sufficiently interpreted.^4^ It may be thought that we need to talk about particles (or fields) and their properties in order for the description provided by the quantum state to have (meta-)physical content. So, let’s grant for the sake of argument that indeterminacy concerning the values of physical quantities possessed by elementary physical systems provides a potential case of fundamental metaphysical indeterminacy in EQM.

In the quote above, Lewis isn’t discussing EQM, but rather the *GRW theory*. One crucial difference between the two approaches is that the latter, and not the former, posits physical collapse. In EQM, by contrast, the measurement problem is solved by appeal to decoherence. Very roughly, when a system interacts with its environment, the quantum state describing it decoheres such that interference between components of the superposition becomes negligible—that is, we can treat it as a statistical mixture. It is at this stage that EQM takes the system to exist in multiple distinct quasi-classical branches, each corresponding to a component of the decohered superposition.

We will return to decoherence below, but for now, note that quantum states which have yet to decohere—*coherent* superpositions—seem to present us with the same case as Lewis described above in the context of the GRW theory:

The consequences in the case of the many-worlds theory are very similar to those for the GRW theory. At the microscopic level, things are almost exactly the same: GRW collapses are sufficiently rare for isolated microscopic systems that for all practical purposes such systems behave as if there were no such collapses—that is, as if the many-worlds theory were true. So in the many-worlds theory, too, microscopic systems can have indeterminate properties, where this indeterminacy is primitive and has nothing to do with compositionality or familiar kinds of vagueness. (Lewis , 2016, p. 97)

So, do coherent superpositions establish the existence of fundamental metaphysical indeterminacy in EQM? We think not, for the simple reason that there are plausible ways of understanding such states that are free from indeterminacy. Notice that in the first quote Lewis moves from lacking a determinate value of a physical quantity to indeterminacy. But, the notions are conceptually distinct. There are several different accounts of metaphysical indeterminacy but they all can be differentiated from simply lacking a given property. This reveals logical space for a view in which systems in a coherent superposition simply lack certain physical properties altogether rather than possessing them in a way that involves indeterminacy.

For example, if we prepare a particle in an eigenstate of spin along some direction *x*, we end up with a superposition with respect to the spin along some orthogonal direction *y*: $\psi = \frac{1}{\sqrt{2}}| \uparrow _y\rangle \pm \frac{1}{\sqrt{2}}| \downarrow _y\rangle$. Prior to decoherence, Lewis would claim that this state describes a particle with indeterminate *y*-spin.^5^ But, there is an alternative given by the *sparse view* (Glick, 2017): The system lacks *y*-spin at this time. On the sparse view, the properties of the system at the time when its quantum state is a coherent superposition of *y*-spin values determinately do not include the property of *y*-spin: it simply does not instantiate any *y*-spin property.

Glick (2017) offers the sparse view as an alternative understanding of *orthodox* quantum mechanics, which posits measurement-induced collapse to an eigenstate. In the context of EQM, where this is not the case, the view needs to be modified as it would seem to imply that there never is any situation in which properties can be ascribed.^6^ Our use of the view here is more general: whenever the advocate of indeterminacy declares that a quantity is indeterminate, there is a sparse alternative which simply jettisons the property. In the present case—a particle in a superposition of *y*-spin which has yet to decohere—the indeterminist finds indeterminacy in the particle’s *y*-spin, and so the sparse alternative rejects that the particle possesses *y*-spin at that time. This is to make no commitments about what happens when there *is* sufficient decoherence for branching. Presumably, at this point, both views would agree that the particle possesses a (determinate) value of *y*-spin on each branch.

Now, if one adopts the sparse view and eschews fundamental indeterminacy, what is left to say about a system described by a coherent superposition in EQM? After all, if the system determinately lacks the physical quantity in question, it may seem that we cannot appeal to that quantity in describing the state of affairs. In his book, David Wallace simply says that “[i]n situations where [decoherence] does not occur, such as the two-slit experiment, there is no parallel-Universe description to offer of the interference process: there is just a quantum system in a very nonclassical state” (Wallace , 2012, p. 382). It may be that there is no adequate ‘classical’ description of such systems, so that all we can say of the system is that it is described by a quantum state that is a particular superposition of the quantity in question. So, it is not the case that we can say nothing about a particle in an eigenstate of *x*-spin with respect to its *y*-spin, but rather, we can only say that it is in a particular superposition of *y*-spin values where this is not to be understood in terms of familiar modes of property possession (including those that engender indeterminacy).

Later we will consider Wallace’s functionalist approach to emergent ontology (Wallace, 2012). What would happen if we were to apply that approach here? Presumably, the particle would not fulfill the functionalist role associated with either *y*-spin up or *y*-spin down. The indeterminist would find indeterminacy in this situation. But the sparse alternative would simply reject that the particle has the property of *y*-spin altogether at this time.

We don’t pretend to have settled the question of fundamental metaphysical indeterminacy in EQM here. We agree with Lewis that the situation is very similar to that encountered in other approaches to quantum theory, and hence, the question will have to be settled in the context of the larger debate over quantum indeterminacy.^7^ That being said, the case of coherent superpositions certainly does not establish that there is metaphysical indeterminacy in EQM, given the existence of alternatives like the sparse view. If we wish to move the debate over quantum indeterminacy forward, we should consider putative instances of indeterminacy that are *unique* to EQM.

## Derivative indeterminacy

Decoherence is central to the contemporary version of EQM. This process is what gives rise to the branches that allow EQM to solve the measurement problem. But, the process of decoherence differs from pure ‘fission cases’ discussed by philosophers in two important respects. First, a plausible understanding of decoherence does not determine a precise number of branches. Second, decoherence does not result in branches that are strictly causally isolated from each other. Each of these differences may be thought to introduce indeterminacy into EQM.

### Decoherence

Decoherence plays two important roles in EQM. First, it provides a preferred basis in which to express the universal quantum state. Second, it ensures that interference between components of the universal quantum state is negligible. By playing this dual role, decoherence allows the Everettian to find a multiverse of quasi-classical branches in the universal quantum state. While we refer readers elsewhere for a full treatment, here we give a brief informal sketch of how decoherence serves these functions.^8^

According to EQM, reality is fundamentally described by the universal quantum state, which takes the form of an extremely complex superposition. Branches are then associated with terms in this superposition state. However, the same quantum state can be represented in a variety of bases, and a change of basis can radically alter the form of the state. So, what branches there are is sensitive to the choice of basis, which seems purely conventional. Decoherence helps to solve this problem by demonstrating that only certain bases give rise to the kind of stable and robust structure required for EQM. For example, consider performing a measurement on a single particle. Suppose that the particle begins in a superposition of the quantity we are measuring, and after the measurement, takes a particular value of that quantity. But what happens if the particle interacts with other particles in its environment on the way to the detector? Each of the particles with which it interacts will effectively ‘measure’ the particle by acting as a record of its position. Decoherence models how the environment constantly measures systems in the position basis in this way. An important caveat here is that the position basis selected by decoherence is only approximate, a point to which we will return shortly.

So, how does decoherence work? Again, consider a single particle interacting with other particles in its environment. If the initial state of the particle is some superposition, then it will linearly evolve into another superposition, in which particles in the environment are entangled with it. For example, suppose the initial state is a superposition of *x*-spin up and down: $\Psi _0 = c_1|\uparrow _x\rangle + c_2|\downarrow _x\rangle$. When the system interacts with its environment it becomes entangled: $\Psi _1 = c_1|\uparrow _x\rangle |E_1\rangle + c_2|\downarrow _x\rangle |E_2\rangle$, where $E_i$ is some specific state of the particles in the environment. Crucially, this state is still a superposition, not a mixture, meaning that characteristically quantum interference could occur between the two components (representing *x*-spin up, environment in $E_1$ and *x*-spin down, environment in $E_2$, respectively). However, decoherence describes how interaction with the environment allows us to view the system as effectively being in a mixture of the two ‘classical’ states. That is, the interference between the terms of the superposition becomes negligible so that we can ignore the quantum nature of the state. For instance, we can ignore (for all practical purposes) the possibility of interference effects once a state has decohered.

The foregoing sketches a way in which decoherence can locate a branching structure in the universal quantum state: environmental interaction approximates constant position measurements and, in the process, interference between components of the superposition (effectively) vanishes. But, how does decoherence give rise to branches which persist over time? To answer this question, it’s helpful to consider the decoherent histories formalism.^9^ So far, we have been thinking in terms of the Schrödinger picture, where the quantum state (or wavefunction) changes with time and the operators associated with physical quantities (observables) are time-independent. If we switch to the Heisenberg picture, this is reversed: states are time-independent and operators evolve with time. On this view, a history $\alpha$ can be represented by an operator $\hat{C}_\alpha$ that is a sequence of projection operators, each of which represents a property of the entire universe:

1 $$\begin{aligned} \hat{C}_\alpha = \hat{\alpha }_1 \ldots \hat{\alpha }_f, \end{aligned}$$

where each $\hat{\alpha }_i$ is a different projection operator at a time. In this setting, we can define the *decoherence functional* between two histories $\alpha$ and $\beta$ as:

2 $$\begin{aligned} \mathcal {D}(\alpha ,\beta ) = \langle \psi | \hat{C}^\dagger _\alpha \hat{C}_\beta | \psi \rangle , \end{aligned}$$

where $\psi$ is the initial quantum state of the universe.

Now, in general, the decoherence functional will be non-zero, which makes it impossible to assign coherent probabilities to the histories.^10^ This represents non-negligible interference between the histories. Decoherent histories are a subset of histories for which the decoherence functional $\mathcal {D}(\alpha ,\beta ) = 0$ for any incompatible (i.e., non-overlapping) histories $\alpha , \beta$.^11^ Two points are worth noting: First, decoherence does not select a unique history but, rather, a decoherent history space, which is a family of histories that are decoherent in the sense described above. Second, the projection operators in a decoherent history will be a *coarse graining* in the sense that they distinguish some physical degrees of freedom and ignore others. This means that even the space of decoherent histories will not be unique; what histories are possible will depend on the fineness or coarseness of the grain.^12^ Thus, “as we fine-grain our decoherent history space, we will eventually reach a point where interference between branches ceases to be negligible, but there is no precise point where this occurs” (Wallace , 2012, p. 100).

### Branch number

Given the nature of decoherence, EQM cannot straightforwardly maintain that there is a precise number of branches in the emergent multiverse. The number of branches is sensitive to how finely grained the history space is, which is conventional within certain limits. As Wallace says, “the question ‘How many branches are there?’ does not, ultimately, make sense” (Wallace , 2012, p. 100). What lessons should we take from this for indeterminacy in EQM?

One possible reaction is to regard the number of branches as metaphysically indeterminate. On this view, perhaps the proposition ‘there are *n* branches’ lacks a determinate truth value for some values of *n*, or the state of affairs involves the instantiation of the determinable *branch number* without it possessing a unique determinate (i.e., a precise number of branches). Crucially, if this is to count as metaphysical indeterminacy, our inability to answer the question ‘how many branches?’ cannot be an epistemic or representational limitation but rather must be a feature of reality itself.

However, this strikes us as an unnatural way to proceed. Typically in physical theorising, if we encounter a quantity that depends on a conventional choice, we regard it as an artefact of the representation rather than an element of reality.^13^ Familiar examples include the choice of an inertial frame in Newtonian mechanics or the absolute values of potentials in classical electromagnetism.^14^ If these quantities are representational artefacts, then the indeterminacy they engender is also representational. It’s not that reality is somehow indeterminate with respect to the values of electromagnetic potentials; rather, they aren’t part of reality. This suggests *eliminativism* about potentials in the context of classical electromagnetism. Below, we will consider a reductionist alternative, according to which the reduced ontology is ‘nothing more’ than the fundamental ontology, differently described. In this case, that would mean that potentials are nothing but a way of describing electromagnetic fields.

Now, in the present case there are *constraints* on the grain of decoherence we adopt. Too fine a grain will induce excessive interference that precludes quasi-classical branches and too coarse a grain could eliminate branching altogether. But, within these broad constraints, there is considerable freedom in our choice. There are many ways of precisifying decoherence, each of which leads to a different number of branches. The same is true of inertial frames and electromagnetic potentials: an inertial frame must satisfy Newton’s first law and an electromagnetic potential must take the form of a four-vector assigned to a spacetime point.

One difference between the cases is that the constraints imposed by decoherence are vague—there is no precisely-defined range of acceptable decoherence bases (or ‘grains’).^15^ This may introduce further issues related to higher-order vagueness, but doesn’t affect our main point. After all, we are considering the selection of a specific basis (or ‘grain’) that meets the decoherence condition *approximately*, so in addition to the conventional choice of a basis, there will be a further conventional choice of what counts as a basis for which the decoherence functional is *close enough* to zero. As with the choice of basis, the vagueness associated with the boundaries of the range of acceptable bases has the hallmarks of representational vagueness: it admits of borderline cases, depends on context, and can be precisified away. That said, if one finds metaphysical indeterminacy in the many cases with this character, one may find it here as well. Our point is that standard physical theorizing regards such conventionality (twice over, in this case) as telling us something about how we are representing the world rather than revealing its true nature.

In light of these prima facie considerations against the claim, is there any basis for regarding indeterminacy in branch number as metaphysical? Calosi and J. Wilson (2022) offer some brief suggestions in a footnote:

...first, it would be more systematic to treat indeterminacy in world number and in world nature similarly; but it is not plausible to treat the lingering indeterminacy in world nature as semantic. And second, a semantic supervaluationist treatment of indeterminacy in world number along lines of the ‘preferred precisification’ approach of McGee and McLaughlin (1995) is subject to the same difficulties that Wilson highlights with an epistemic approach to indeterminacy—namely, that it presupposes, implausibly, that there is a single preferred precisification/basis/decoherent history space/multiverse. (Calosi & J. Wilson, 2022, footnote 8, p. 381)

We concur that, indeed, it is desirable to treat branch number and branch nature similarly. However, whether they should be both interpreted as involving metaphysical indeterminacy remains an open question. We will show below that there are perfectly legitimate ways to take both indeterminacy in branch number and branch nature to be representational. Regarding the second point about branch number, one need not presuppose there is some *privileged decoherent history space* (or ‘grain’) to make sense of representational indeterminacy in branch number. If the number of branches is a representational artefact, then, as Wallace says, there is simply no answer to the question of how many branches there are. Like the absolute value of electromagnetic potentials, it is indeterminate precisely because it is the result of a conventional choice in how we represent the physics.

With respect to the epistemicist view, we disagree somewhat about what’s problematic with the claim that there is a precise number of branches. The main issue here is not that the epistemicist view is implausible, or at least it’s not clear why exactly epistemicism is supposed to be implausible. The existence of a single preferred decoherent history space would be a brute fact, as epistemicism as a general strategy in various contexts, must rely on the existence of brute, arbitrary facts. And it is a good methodological principle to avoid such arbitrary facts—this is perhaps what Calosi and J. Wilson mean when they take epistemicism to be ‘implausible’. In our view, a more compelling worry is that the brute unobservable facts the strategy requires are undesirable in the context of EQM, which bases its ontology directly on the formalism. The formalism makes no reference to branches, and so to no fixed number of branches.^16^ This nuance aside, we agree with Calosi and J. Wilson that a single preferred decoherent history space is undesirable.

However, and this will be the crux of our argument, the lack of a single preferred decoherent history space does not necessarily entail that the number of branches is metaphysically indeterminate. Again, given that it is the result of a conventional choice of particular history space, it is natural to regard the indeterminacy as representational in nature. One way to make sense of this is to posit an unknowable preferred history space, and take the indeterminacy to be epistemic. But, a more attractive option is to regard the use of the decoherent histories formalism to introduce representational artifacts and, as we shall see, to either reduce or eliminate branches from our ontology.

### Branch nature

Indeterminacy in branch nature results from the fact that decoherence is imperfect so that Everettian branches are never perfectly isolated from each other. After a process of decoherence, there will remain some residual interference between branches, however negligible.^17^ This is why branches are merely *quasi-*classical. The residual interference between branches means that the nature of branches, even when described at a coarse-grained level, may depart from classical expectations. For example, a pointer may determinately indicate a particular measurement result on a branch at one time, but may change or disappear at a later time without any intra-branch cause. Rather, the pointer’s location could change as a result of the influence of another branch. This makes it natural to think that there is a lack of determinacy in the location of the pointer, even after decoherence has suppressed interference between branches.

Moreover, macroscopic objects like pointers will not have precise positions on the decoherence basis, even within a branch. Rather, they will be in a position state that is strictly a superposition of precise positions, even though it is sufficiently close to an eigenstate of position that it may be treated as one for all practical purposes.^18^ As Calosi and J. Wilson say, “decoherence does not eliminate *all* interference between components of a given superposition, and hence the values of the associated observables in the state components are rendered only comparatively or relatively determinate—that is, to some small extent indeterminate” (2022, p. 383).

A. Wilson (2020, p. 181) illustrates the indeterminacy of branch nature with so-called ‘Schrödinger kittens’—experiments where relatively large systems are carefully prepared in coherent superposition states (Arndt & Hornberger, 2014). While interesting, such cases fail to establish metaphysical indeterminacy on our view. As discussed above, such systems may be regarded as simply lacking the properties appearing in the terms of the superposition rather than possessing indeterminate values of them (*à la* sparse view). However, this same approach doesn’t carry over to the properties of systems described by a quantum state that has decohered. For such systems, the sparse view would suggest that they lack nearly all properties given that the contents of branches are not strictly described by eigenstates. But, this is problematic—the Everettian should be able to say that a measurement has a determinate result in each branch. Fortunately, one can deploy more or less forgiving principles linking the quantum state to familiar physical properties. In particular, *functionalist* principles tend to be forgiving in this sense. So, if we functionally characterize the pointer as having a precise location in a branch, we can say that its location is fully determinate, even though it’s not strictly in an eigenstate of the observable associated with that location, provided that *it behaves like an object with that location*. The cost of doing so is to introduce indeterminacy in the functional definition—the italicized phrase above—but such indeterminacy is familiar from ordinary cases of vagueness and amenable to the usual deflationary treatments. For instance, if we precisify the phrase in question, the indeterminacy vanishes.^19^

We acknowledge that derivative metaphysical indeterminacy is a theoretical possibility in this context, provided one adopts the right linking principle between fundamental and derivative ontology. However, we will maintain against Calosi and J. Wilson (2022) that a *deflationary linking principle* is plausible in this context, and that indeterminacy in branch nature should not be viewed as anything more than merely representational indeterminacy.

## Inter-level links

Given that there is no decisive reason to posit fundamental indeterminacy in EQM, the argument in favor of metaphysical indeterminacy will turn on how the fundamental level relates to derivative levels. We call the principle linking the fundamental to the derivative an *inter-level link*. One candidate for such a link is the eigenstate-eigenvalue link, which establishes the existence of an observable with a particular value when (and only when) the quantum state is an eigenstate of the associated operator.^20^ To qualify as an inter-level link, the observables attributed would have to be understood as existing at a less fundamental level than the quantum state. It doesn’t matter for our purposes whether this is the *correct* understanding of the eigenstate-eigenvalue link, which we use merely as an illustrative example of a possible inter-level link. This particular link is implausible in the context of EQM for the reason given above: decoherence fails to secure stable eigenstates, even within a branch. Wallace adopts a functionalist link for higher-level ontology, and we agree that this is well-suited to EQM. The important point is that any such link, which connects the quantum state to less fundamental ontology, can be understood either in an inflationary or deflationary manner, with different results for the question of metaphysical indeterminacy in EQM.

### Inflationary

First, consider inter-level links that are inflationary and determinacy-preserving. Such links are inflationary in the sense that they commit one to the existence of derivative entities that are distinct from the fundamental ontology, and determinacy-preserving in that they preclude the possibility of fundamental determinacy and derivative indeterminacy co-occurring. This seems reasonable: If facts about the fundamental level *entail* facts about derivative levels, then it seems that merely derivative indeterminacy is impossible (for a full-blown defense of this point, see Barnes , 2014).

Are the links relevant to EQM determinacy-preserving? First, consider the simpler case of the eigenstate-eigenvalue link. This link takes the form of a biconditional between the quantum state and the ascription of a physical quantity taking a particular value. This certainly looks to be determinacy-preserving; assuming the quantum state is fundamental and determinate, the eigenstate-eigenvalue link will provide a fully determinate answer to the question of whether or not there is a physical quantity taking the value in question. Thus, derivative indeterminacy only follows if the eigenstate-eigenvalue link is revised (Calosi & J. Wilson, 2021) or extended to properties beyond simple physical magnitudes.

Given that the eigenstate-eigenvalue link is implausible in EQM, what is the inter-level link between the fundamental quantum state description and the derivative multiverse description? For Wallace (2012), it’s functionalism; for A. Wilson (2022), it’s grounding. Either could be understood as determinacy-preserving. If the functional role is precisely specified, then the situation is no different than the eigenstate-eigenvalue link; we have necessary and sufficient conditions for derivative facts that are free from vagueness. Regarding grounding, as Barnes notes, “if you think the fundamental facts ground the derivative facts then, provided that ‘Determinately, p’ and ‘p grounds q’ entails ‘Determinately, q’, the argument still works, and still rules out the situation whereby we have determinate fundamental reality that gives rise to an indeterminate derivative reality” (*op. cit.*, pp. 342–343). Indeed, one might wonder whether non-determinacy-preserving links are possible at all.

However, as argued by Mariani (2022), one can resist Barnes’ argument by adopting a different account of metaphysical indeterminacy. In particular, on the determinable-based account of metaphysical indeterminacy due to J. Wilson (2013), a state of affairs is indeterminate just in case it involves the instantiation of a determinable property without the instantiation of a unique determinate of that determinable. Such an indeterminate state of affairs could obtain at a non-fundamental level while the fundamental level is perfectly determinate.^21^ For our purposes here, we are happy to grant that such a situation is possible. That is, we can have a link that is both inflationary and fails to preserve determinacy.

In the context of EQM, such a link would combine a fully determinate fundamental level described by the universal quantum state with a derivative level that consists of an indeterminate number of branches, each of which includes some indeterminate properties. For instance, the link would say that even after suitable decoherence occurs, the position of a pointer will have an indeterminate location in virtue of lacking a unique precise position within a branch.^22^

Such an inflationary attitude toward derivative ontology, coupled with the denial that the link between levels must preserve determinacy, provides in our opinion the most compelling case for metaphysical indeterminacy in EQM.^23^ However, arguments of this type do not rely solely on the physics of EQM, they require another layer of metaphysical interpretation. But, as we shall see, there exist alternative metaphysical interpretations of EQM that are more in harmony with the spirit and methodology of EQM, and fit nicely with the natural view that the indeterminacy in the number of branches is representational rather than metaphysical.

### Deflationary

Above we claimed that a natural attitude to adopt about branch number is the deflationary view that because branch number depends on a conventional representational choice, we shouldn’t view it as an aspect of reality. Can we adopt a similar deflationary attitude toward branch nature in EQM? Indeed we can. On the deflationary view, any indeterminacy introduced by the multiverse description is representational in nature, and can be eliminated by descending to the more fundamental description. We grant that this attitude is somewhat revisionary given recent presentations of EQM (Wallace, 2012; A. Wilson, 2020). But it’s not without precedent.

Wallace (2002), for example, develops an extended analogy between branches in EQM and instants of time in relativity. The overarching claim is that both provide a higher-level description from which one can recover the more fundamental description, but which contains a certain amount of conventionality. We will develop the point below, but note already that a deflationary attitude toward global instants (described as three-dimensional Cauchy surfaces) seems reasonable, insofar as there exist multiple ways to slice a four-dimensional relativistic spacetime into those slices. This is an instance of a general deflationary attitude we favour also in the case of EQM, and which, we will see, is also favoured by Wallace (2002). Thus, we grant that the link between the universal quantum state and the multiverse introduces indeterminacy, but we regard this indeterminacy as representational in light of the fact that the multiverse description is—like any description of spacetime given a particular foliation—a representation that is less than fully perspicuous.

Such a deflationary approach to EQM branches has several advantages on the face of it. First, the ontology is more parsimonious than the inflationary approach, because it doesn’t posit branches as entities distinct from the universal quantum state. Second, there is no need for a theory of metaphysical indeterminacy—of course, this consequence will not necessarily appear as an advantage, depending on which side of the fence the reader is leaning. Third, the approach fits harmoniously with the natural understanding of the indeterminacy of branch number. Holding the same view on the indeterminacy of branch nature and branch number avoids the delicate problems posed by hybrid views that would consider one of the two phenomena to be purely representational, and the other metaphysical, when they are not independent of each other. In brief, since it’s so natural to be a deflationist about the indeterminacy involved in branch number, we get a strong incentive to also be deflationist about branch nature.

### Eliminativism or reductionism?

How are we to develop a deflationary view with full ontological clarity and does it require saying that branches in EQM aren’t real? To begin addressing these questions, consider Wallace’s (2002) suggestion that we understand the universal quantum state as

...an entity with such-and-such mathematical description *which can be thought of as a collection of instantaneous worlds together with their Hilbert-space amplitudes, no matter that it may be decomposed into such a collection in many different ways.* (Wallace , 2002, p. 647, original emphasis)

On this view, branches provide a *way of understanding* the fundamental ontology of EQM, the universal quantum state. That the decomposition into branches pertains to an understanding and not directly to the fabric of reality paves the way for a deflationary explanation. Not everyone will agree that relegating branches to being a tool for understanding will avoid ontological commitment but we follow here Woodward (2005) and De Regt (2017) who defend that “understanding is not a matter of getting the ontology right” (De Regt , 2017, p. 65).^24^ There are two ways to gloss this deflationary approach: *eliminativism* and *reductionism*.

Perhaps most naturally, we can be eliminativists about Everettian branches; they are a useful conceptual tool, but not elements of reality. The concept of an Everettian branch would thus offer a means to comprehend the ontology of quantum mechanics, but not in a way that is ontologically committing. Alternatively, one can endorse reductionism about Everettian branches by regarding them as ‘nothing but’ parts (or aspects) of the quantum state under a different description. Both offer viable ways of understanding EQM without positing metaphysical indeterminacy.

To better appreciate the two options, it is instructive to dwell on the question of the existence of instants in the context of relativistic physics that we mentioned before, following (Wallace , 2002, pp. 641-643). There are many ways to carve spacetime into instants. Does this imply that instants do not exist? In a certain sense yes, if we require for instants to exist that spacetime is uniquely and privilegedly decomposable into three-dimensional surfaces. In another sense no, if we do not require that instants correspond to a unique privileged decomposition of spacetime into three-dimensional surfaces. This ambiguity about the appropriateness of adopting the uniqueness of the privileged decomposition as a constraint leads to the two different strategies of reductionism and eliminativism applied to instants in relativity. The situation is similar with the decomposition of the universal quantum state into branches in EQM. That there is no unique, privileged, decomposition of it into branches, leads likewise to the same two strategies. Let us examine them in turn.

Consider, first, the motivation for eliminativism about branches. This view, while certainly revisionary (EQM is known as the ‘many-worlds theory’), has certain benefits. Much of the motivation for EQM stems from its adherence to unmodified unitary quantum mechanics. There is a certain simplicity in the Everettian limiting their ontological commitments to those found directly in the formalism. And there is no reference to branches to be found in the formalism. Thus, one should eliminate branches from the ontology of EQM. A possible cost in doing so is that many other entities do not appear in the formalism to which we, nonetheless, want to grant existence. Think, for instance, about the Milky Way or ordinary objects like tables and chairs. They don’t appear directly in the formalism either, so one might argue that the requirement to accept the existence of entities that are explicitly mentioned in the formalism is too strict. Just as ordinary objects exist without appearing in the formalism, so do branches.

There is, however, a flaw in this argument, which is to accept from the outset that we should grant the existence of ordinary objects—and more generally, of all common-sense entities that do not appear in the formalism of quantum mechanics—as a primitive fact. It’s a general strategy in metaphysics to eliminate common-sense entities for various reasons.^25^ But note that here the task is even simpler, since we never really observe anything as a branch—indeed, this concept is primarily theoretical, with no experiential basis, unlike the concept of ordinary object. Moreover, and this is the crucial point in this case, if the main motivation for postulating the existence of branches is the formalism itself, that the formalism makes no mention of these branches should at least give us pause. This fact seems to be a reasonable motivation for asking whether those branches exist.

The main problem with branch eliminativism, at least at first sight, is the concern that we need branches, or at least our own branch, to make sense of our experience of physical reality. It now becomes important to circle back to the distinction between branches and worlds. As mentioned before, we distinguish between local branches, and global worlds. A branch, in this sense, corresponds in the relativistic conceptual apparatus to a growing lightcone. When decoherence induces the branching, a lightcone gets generated with the propagation of the electromagnetic signals that plays the decoherence role. Note this is a simple heuristic consistent with a four-dimensional, block-universe picture, and with different views regarding whether branches split by diverging or overlapping (A. Wilson, 2012). What matters is that the newly born branch is confined to a lightcone structure and its interior: the newly born structure doesn’t range over spacelike hypersurfaces outside the past lightcone.

Worlds are more inclusive structures. Unlike branches, they convey a notion of totality. A world, unlike a branch, encompasses not only the interior of the past lightcone, but also the rest of the spacetime, including the regions sometimes called ‘elsewhere’. Recall that the branching is induced by decoherence, a local process bounded by the speed of light. One could then ask whether branching—being a local process—merely generates local branches, or global worlds exceeding the lightcone bounds. Arguably, getting a better grip on this question requires a foray into the study of the possibly quantum nature of the metric field, and hence assessing this question in the context of various approaches to quantum gravity. Here is not the place to argue for one of the other of the two interpretations. But they give us a richer understanding of what the question of the reality of branches and worlds really amounts to, by emphasising some crucial ambiguity on what is meant by the concept of branch or world in the first place.

For consider, first, the notion of global worlds. It should be clear that worlds, in this sense, lie beyond empirical observation. By including events that are spacelike separated from us, they posit non-directly-observable events and their nature remains somewhat theoretical. Thus, one can never observe the world in which we are located, in this sense. At best, one can observe a (fairly small) subregion of our world. Of course, one could immediately reply that what we have evidence for is not the existence of our (global) world but only, more modestly, of the (local) branch to which we belong. But, again, we do really observe something like a branch? We believe not: we no more observe branches than we do observe worlds. What we observe is a small proper part of our branch, mostly mediated by electromagnetic signals.^26^ Although we are causally connected to our past lightcone under a certain conception of causation, we lack direct visual access to most of our past lightcone, namely the set of events timelike separated from us. After all, we merely see the material events that intersect with the past lightcone’s boundary. Furthermore, as decoherence happens at the speed of light, it implies the existence of events located in our own future lightcone, to which we lack empirical access and that, arguably, should be legitimately viewed as proper parts of our branch.

It should thus be clear that we have no empirical access to our whole branch or our whole world. But, then, what do we really need to account for in order to make sense of our empirical experience and common-sense picture of physical reality? This is related to a larger question of empirical coherence in quantum mechanics concerning the possibility of doing physics in an apparently spatial branch if space does not exist (Barrett, 1996; Ney, 2021), or in a non-spatiotemporal universe in the context of quantum gravity (Huggett & Wüthrich, 2013). However, the debate has largely been resolved insofar as it is now commonly accepted that there is no problem of empirical coherence as long as we can recover the possibility of doing physics in a very practical way by using concrete instruments such as rods and clocks. Recovering somewhat general theoretical structures such as space, time, or spacetime, is not needed to solve the problem of empirical coherence (Baron & Le Bihan, 2022).

What matters, similarly, in the context of EQM, is the possibility of using rods and clocks to make measurements and, more generally, accounting for the relevant structures needed to make sense of the practice of physics in our immediate surroundings. And as we have seen, decoherence and functionalism can explain how we can use and refer to parts of the universal quantum state in a seemingly classical, but ultimately quasi-classical, way. Thus, eliminativism seems well suited to the ontology of EQM, on the whole.

Now, consider the chief alternative to eliminativism: *reductionism*. The branches are *reducible* to the universal quantum state but remain nonetheless real. More precisely, the concept of an Everettian branch picks out aspects of the universal quantum state. Branches, themselves, exist in being numerically identical to parts of the universal quantum state. Again, consider the analogy with global times in relativity. As we have pointed out, there are many possible ways to carve out three-dimensional hypersurfaces corresponding to global instants in relativistic spacetimes of the right kind, and we submitted that this gave incentive to reject global instants. Yet one could equally admit the existence of these global times, arguing that they are reducible to slices of spacetime—even though the slicing can be performed in numerous different ways.^27^

The same could be said, in principle, of the universal quantum state in EQM. The branches would exist, but not as the result of a single decomposition. They would be reducible to slices of the universal quantum state. There are a number of different accounts of ontological reduction in the literature, but a common idea is that an entity is reducible just in case it is ‘nothing but’ some lower-level entity or entities, differently described. In the present case, this would amount to branches being nothing but (parts of, or aspects of) the universal quantum state. This fits nicely with the ‘can be thought of’ language in the passage from Wallace. Moreover, this would mean that any indeterminacy engendered by branches is merely representational—a result of the description of the quantum state adopted. It’s worth taking a moment to clarify how this works for each potential source of derivative indeterminacy in EQM.

The putative indeterminacy of branch number results from the lack of unique decoherence grain or history space. For the eliminativist, there is no such indeterminacy because there is in fact a determinate number of branches: 0. For the reductionist, this is a plausible case of indeterminacy insofar as there are branches, but there is no way to say how many, because different choices of grain yield different answers. Indeed, according to J. Wilson’s determinable-based account, this would count as metaphysical indeterminacy in which multiple determinates of the determinable *branch number* are instantiated, each relative to a different decoherent history space. However, as mentioned above, regarding this indeterminacy as metaphysical is in tension with the fact that the choice of a history space is largely conventional, and hence, shouldn’t have an effect on reality. As Wallace says, ‘how many worlds are there?’ is a non-question—it rests on a misunderstanding of the kind of thing branches are:

Put another way: asking how many worlds there are is like asking how many experiences you had yesterday, or how many regrets a repentant criminal has had. It makes perfect sense to say that you had many experiences or that he had many regrets; it makes perfect sense to list the most important categories of either; but it is a non-question to ask how many. (Wallace , 2012, p. 102)

In sum, when it comes to branch number, the reductionist will grant that there is indeterminacy involved, but argue that it’s merely representational given that it results from a description of quantum reality that is less than fully perspicuous. The number of branches is an artifact of the representation even if the branches themselves are not.^28^

Now, turning to branch nature, recall that the issue here is that Everettian branches aren’t strictly isolated from one another, and hence even macroscopic systems may not be in stable eigenstates of operators corresponding to physical quantities on a single branch. Again, the eliminativist will straightforwardly deny there is any indeterminacy involved. If there are no branches, and no macroscopic entities, then there is simply no way to get the argument for indeterminacy going. For the reductionist, however, macroscopic objects and branches exist, and there is no way to eliminate the residual interference between branches that motivates indeterminacy in branch nature. But, again, the reductionist will urge that the indeterminacy is representational rather than metaphysical. As noted above, there is no automatic inference from a quantum state that is not an eigenstate of an operator associated with a physical quantity to indeterminacy concerning that quantity. For instance, on the functionalist approach, we may allow that a physical system possesses, say, *location in region R* despite its position wavefunction lacking compact support in *R*. Now, this will engender indeterminacy insofar as functionalist criteria of this kind admit of borderline cases, but this indeterminacy is naturally understood as representational. If we precisify the functionalist description, any indeterminacy will be eliminated—either the system determinately will (will not) be located in *R* or not. So, the reductionist story here is much the same as in the case of branch number: Macroscopic objects are real, and possess physical properties within a world, but it may be indeterminate which properties they possesses given that our functional characterization of those properties is vague. This only provides a case of metaphysical indeterminacy if one regards the vagueness involved in such functional criteria as more than merely an aspect of our representation but EQM provides no new reasons for this claim.^29^

## Conclusion

Whether the indeterminacy involved in branch number and nature is metaphysical depends on the nature of the inter-level link deployed. Deflationary readings are available on which the indeterminacy involved is merely representational. Eliminativism and reductionism about branches offer two alternative deflationary readings, and come with different prices. Eliminativism is a fairly radical view, as it denies the very existence of branches. Reductionism is also radical in a certain sense, by relativising branches to alternative decompositions of the universal quantum state. But both are in line with the standard scientific methodology that usually regards indeterminacy engendered by conventional choices to be representational. Overall, we think that the proponents of metaphysical indeterminacy in EQM are too quick in abandoning one of the original motivations of EQM, which was to replace indeterminacy with multiplicity. Note that, strictly speaking, the eliminativist view doesn’t preserve multiplicity either—since the multiplicity of branches does not exist. Eliminativism leads to a monist view on which the universe is just one universal quantum state. Nevertheless, the view preserves the spirit of the original motivation in that the best way to *understand*, in an idealised way, the ontology of the universal quantum state is in terms of multiplicity—not of indeterminacy.

Irrespective of whether one opts for eliminativism, reductionism or an inflationary view, this choice will be based on considerations mostly external to EQM. Indeed, the quantum formalism makes no mention whatsoever of worlds or branches, and hence, indeterminacy of branch number and nature can’t be grounded in EQM alone: further metaphysical principles that go beyond the physical theory are needed. Absent an argument for such principles, one is free to conclude that there is no indeterminacy (on the eliminativist view), or that it’s the result of our choice of representation (on the reductionist view). Those who claim to find new evidence for metaphysical indeterminacy in the picture given by EQM, need to rely on metaphysical assumptions (e.g., an inflationary inter-theory link) that are in tension with the idea that EQM is just unitary quantum mechanics, literally understood (Wallace , 2012, p. 2). This assumption should motivate us to move away from the inflationary view, and to reject altogether the existence of derivative metaphysical indeterminacy in EQM.

Combining this result with that of Section 2—namely, that there is no novel argument for fundamental indeterminacy in EQM—we arrive at the conclusion that EQM does not provide any new reasons to believe in metaphysical indeterminacy. Of course, this is not to say that one couldn’t posit metaphysical indeterminacy in the context of EQM, but this would be true of nearly *any* physical theory. Physics underdetermines metaphysics, but naturalism recommends taking the former as the guide to the latter. In the present case, we have shown that there are natural ways of understanding EQM without introducing metaphysical indeterminacy.

## Footnotes

1 In our terminology, *branches* are local, while *worlds* are global. On the local/global distinction, see, e.g., Waegell and McQueen (2020). Our notion of local branch corresponds roughly to the notion of local world in the parallel lives literature (see Waegell , 2018). If the branching is a local process that occurs at the speed of light in EQM, as commonly assumed, then there is a substantive question regarding whether the branching into local branches also generate relativistic spacetimes in their entirety, namely global worlds in our terminology. We will set aside this question in the present paper but will circle back to the distinction between branches and worlds when discussing perceptual arguments in favour of the existence of branches and worlds. Note also that we will talk in the present paper of ‘branching’ in a broad sense, which is compatible with both branching in the narrow sense (the branching process generates new branches/worlds that come into existence) and divergence (the branching process signals the pre-existence of branches or worlds that were already there, and were qualitatively indistinguishable yet numerically distinct).

2 See also Wallace (2013, p. 464).

3 On the notions of fundamentality and emergence involved in quantum gravity, see e.g., Huggett and Wüthrich (2013); Crowther (2019).

4 See, e.g., Wallace and Timpson (2010), who argue that the quantum state assigns density operators to regions of spacetime. Wallace and Timpson defend this as an unfamiliar, but adequate account of fundamental reality.

5 We take this to be neutral between different metaphysical understandings of indeterminacy. For example, it could mean either ‘it is unsettled whether the *y*-spin is up (down)’ or ‘it possesses the determinable *y*-spin without a unique determinate of it’.

6 This is because the sparse view countenances properties only when the system is in an eigenstate of the associated operator (in keeping with the eigenstate-eigenvalue link). But, the approximate nature of decoherence fails to establish strict eigenstates of observables, even within a branch. (See Section 3 below.) Thus, applying the sparse view to EQM would force one to jettison nearly all properties corresponding to macroscopic observables.

7 For an overview of the debate, see Calosi and Mariani (2021).

8 See Wallace (2012, chapter 3) for a discussion of decoherence in the context of EQM. For a more general discussion, see Bacciagaluppi (2020) and the references therein.

9 Our presentation follows Wallace (2012, pp. 91–99).

10 The probability of a history $\alpha$ is given by: $Pr(\alpha ) = \langle \psi | \hat{C}^\dagger _\alpha \hat{C}_\alpha | \psi \rangle = \mathcal {D}(\alpha ,\alpha )$. The probabilities of non-overlapping histories $\alpha$ and $\beta$ will only be coherent (with their total sum equal to one) if the real part of the decoherence functional between them vanishes. Two histories overlap just in case they share a time-indexed operator $\hat{\alpha }_i$.

11 Gell-Mann and Hartle (1993) call this condition ‘medium decoherence’. There are several distinct decoherence conditions in the literature. See Wallace (2012, pp. 97–99) for a discussion. For our purposes here, it will be useful to consider spaces of histories which satisfy Eq. 2 only *approximately*. These can be understood as histories with negligible mutual interference.

12 A decoherent history space can be refined by replacing one or more of the projectors that appear in the histories with other projectors that add up to it, and it can be coarsened by adding together two or more projectors to form a new projector and using that to construct a new history space.

13 Note that the fact that conventional choices might be more or less arbitrary, by tracking physically interesting facts in specific contexts, doesn’t matter here. What matters here is the presence and necessity of *conventional choices*, which are not usually taken to signal the existence of metaphysical indeterminacy.

14 There is some room for disagreement about these cases, but the dominant view among contemporary philosophers maintains that Newton was mistaken to think that his laws required a privileged inertial frame sufficient to define absolute space and time. The case of electromagnetic potentials is complicated by the Aharonov-Bohm effect, but if we limit ourselves to applications of classical electromagnetism in *classical mechanics*, the consensus view is that the absolute values of potentials lack physical significance.

15 Thanks to an anonymous referee for raising this concern.

16 As Hilary Greaves, says, the question of how many branches exist “presupposes the existence of a piece of structure that is not in fact present in the theory” (2007, p.120).

17 Recall that the more fine-grained our decoherent history space, the more interference between branches there will be. This connection with branch number indeterminacy tells against a hybrid approach which would regard one form of indeterminacy as representational and the other as metaphysical. It also means that the *degree* of branch nature indeterminacy is indeterminate—it depends on our choice of grain—but branches will contain some degree of indeterminacy regardless of the coarse graining we adopt (see, e.g., A. Wilson , 2020, pp. 180–181).

18 Whatever observable defines the branching will necessarily be in an eigenstate in each branch, but (a) decoherence doesn’t determine a unique basis and (b) even if it did, all other observables would fail to be in eigenstates. For example, if we identify the decoherence basis with the pointer basis, then the pointer will be in an eigenstate in each branch, but the rest of the systems in the branch would not be. And even the pointer may behave in a non-classical manner. For a discussion, see Lewis (2016, pp. 99–101).

19 As a simple example, behaving like an object located in region $\Delta$ requires a high probability of detection in $\Delta$ if measured. How high this probability should be is vague, but could be made precise. Of course, wherever we place the threshold for determinate property possession, there will be systems that fall below that threshold. These might include the sort of Schrödinger kittens mentioned by A. Wilson. But, again, there is no automatic inference from such systems lacking certain determinate properties to the presence of metaphysical indeterminacy.

20 More precisely, according to the eigenstate-eigenvalue link, a system *A* has a value *v* of property *P* iff the quantum state of *A* is an eigenstate of the associated operator $\hat{O}$ with eigenvalue = *v*. For a discussion, see Gilton (2016).

21 The standard example of the determinable/determinate relation is: *scarlet* is a determinate of the *red* determinable. Now, if we imagine that it’s possible for something to be red without being a unique shade of red, this would constitute metaphysical indeterminacy on this view, regardless of the level of reality at which colors exist. Moreover, this would still be the case even if all instances of determinables had unique determinates at the fundamental level (which, presumably, doesn’t contain colors).

22 For example, on the ‘glutty’ degree-theoretic implementation of determinable-based indeterminacy discussed by Calosi and J. Wilson (2021), within each branch the pointer would possess some precise position to a very high degree, but would also possess other precise positions to lesser degrees.

23 An inflationary approach to higher-level ontology in EQM doesn’t necessarily result in derivative metaphysical indeterminacy. For example, consider David Lewis’s (1986) approach to vagueness. On Lewis’s view, there are an abundance of precise objects and vagueness results from semantic indecision about which of these we refer to with our ordinary language terms. One could try to adopt this approach in EQM by positing a *super-inflationary* link that would result in the existence of every acceptable collection of branches—each candidate decoherent history space describes a plurality of actually existing branches. With this abundant ontology in place, indeterminacy of branch number is simply semantic indecision about the referent of our term ‘branch’. This view faces a number of challenges (e.g., higher-order vagueness) and would have to be extended to cover branch-nature indeterminacy, but it’s not obviously untenable. This shows that an inflationary link alone isn’t sufficient for metaphysical indeterminacy in EQM—more work will have to be done by its advocates to argue for its presence in inflationary EQM.

24 One could for instance link understanding to counterfactual consequences. De Regt sums up nicely Woodward’s view of understanding, in relation to his interventionism: “Understanding is not a question of getting the ontology right, but of knowing how systems behave under a range of interventions” (De Regt , 2017, p. 65). De Regt himself defends a more neutral view where understanding can come together, or part company, with ontological assumptions: “On my account, even anti-realists (who do not believe that scientific theories aspire to be true descriptions of the unobservable world) can endorse understanding as an epistemic aim of science” (De Regt , 2017, p. 137). As we mentioned, not everyone agrees as those for whom understanding is necessarily truth-conducive: see, e.g., Khalifa (2020) and references therein.

25 Eliminativism about ordinary objects has been defended for instance by Merricks (2003) and Benovsky (2018).

26 We also perceive events via other senses mediated by other matter fields.

27 Many would say that in the context of a relativistic spacetime that admits of Cauchy surfaces, instants of time exist *relative to a choice of reference frame*. Maudlin is one author who objects to this way of putting things (Maudlin , 2012, p. 92).

28 Another way of making this distinction may draw on supervaluationist approaches to vagueness (see, e.g., A. Wilson , 2020, pp. 173–180). Branch number is indeterminate in that eligible precisifications of ‘branch’ differ about branch number, but all will agree that branches exist. So, it’s determinate that branches exist, but indeterminate how many. Again, such indeterminacy is most naturally understood as representational—if we specify a unique precisification it vanishes.

29 This is not to say that one couldn’t argue for the claim that the indeterminacy engendered by functionalist criteria is metaphysical, but rather, that there is nothing special about the case of EQM in this respect. After all, functionalist criteria for property ascription abound, and inevitably involve a certain degree of vagueness.
